# ADC correlation with Sirtuin1 to assess early chemoradiotherapy response of locally advanced esophageal carcinoma patients

**DOI:** 10.1186/s13014-019-1393-y

**Published:** 2019-11-04

**Authors:** Ying Chen, Tieming Xie, Zhimin Ye, Fangzheng Wang, Dan Long, Mingxiang Jiang, Jun Fang, Qingren Lin, Kai Li, Zhun Wang, Zhenfu Fu

**Affiliations:** 1 Institute of Cancer and Basic Medine (ICBM), Chinese Academy of Sciences, Hangzhou, People’s Republic of China; 2 Department of Radiation Oncology, Cancer Hospital of the University of Chinese Academy of Sciences, No. 1 Banshan East Road, Hangzhou, People’s Republic of China 310022; 3 Department of Radiation Oncology, Zhejiang Cancer Hospital, No.1 of Banshan East Road, Hangzhou, People’s Republic of China 310022; 4 Department of Radiology, Cancer Hospital of the University of Chinese Academy of Sciences, Hangzhou, People’s Republic of China 310022; 5 Department of Radiology, Zhejiang Cancer hospital, No.1 of Banshan East Road, Hangzhou, People’s Republic of China 310022

**Keywords:** Esophageal carcinoma, Diffused weighed imaging, Apparent diffused coefficient, Sirtuin1, Early response

## Abstract

**Aims:**

To determine the biological correlation between apparent diffusion coefficient (ADC) values and Sirtuin1 (SIRT1) levels of tumour tissues in patients with esophageal carcinoma (EC), and to ascertain the treatment biomarker of ADC in predicting the early response of patients undergoing definitive chemoradiotherapy (CRT).

**Methods:**

A total of 66 patients were enrolled, and the specimens of tumour tissues were collected before treatment to perform immunohistochemical (IHC) examinations and quantify the levels of SIRT1. Then all patients were given two esophageal magnetic resonance imaging (MRI) examinations with diffused weighed imaging (DWI) including pretreatment and intra-treatment (1~2 weeks after the start of radiotherapy). The regions of interest (ROIs) were contoured according to the stipulated rules in advance using off-line software, and the values of the ADC in the ROIs were generated automatically. Then, the values of the ADC at baseline and intra-treatment were labeled as pre-ADC and intra-ADC respectively, and ΔADC, ADC_ratio_ were calculated. Pearson’s correlation coefficients were acquired to estimate the correlation between each of ADC values and SIRT1 levels. Spearman’s rank correlation coefficients were acquired to estimate the correlation between early response and the values of each ADC. Receptor operation characteristics (ROC) curves were constructed to estimate the accuracy of the ADC in predicting the early response of CRT.

**Results:**

The findings of this study showed different correlations between ADC values and the levels of SIRT1 (ΔADC: *r* = − 0.943, *P* = 0.002; ADC_ratio_: *r* = − 0.911, *P* = 0.000; intra-ADC: *r* = − 0.748, *P* = 0.002; pre-ADC: *r* = 0.109, *P* = 0.558). There was a positive correlation between ΔADC and early response to treatment (*ρ* = 0.615, *P* = 0.023), and multivariable logistic regression revealed that ΔADC was significantly associated with short-term response of CRT in esophageal carcinoma patients.

**Conclusions:**

In summary, early increases in ADC may facilitate the predication of early CRT response in patients with esophageal squamous cell carcinoma (ESCC), which may be attributed to the different correlation between ADC changes and SIRT1 expression.

## Introduction

Esophageal carcinoma (EC) is one of the most devastating cancer worldwide, including over 450,000 new cancer diagnoses yearly, and in some areas of China, this disease is the fourth leading cause of mortality from cancer, with an esophageal squamous cell carcinoma (ESCC) histology in approximately > 90% of cases [1, 2]. Unfortunately, most patients diagnosed with esophageal cancer are at a locally advanced stage with unresectable or metastatic disease; therefore, concurrent chemo-radiotherapy (CRT) is currently considered the best treatment modality [3]. On the other hand, patients who are insensitive to CRT (18–25%) may be damaged by the toxicity of an ineffective therapy without survival benefit [4]. Hence, if the CRT response is well monitored earlier, the effectiveness of the treatment regimens will be better predicted, and then the early identification of patients who are at higher risk of poor response before CRT would allow for individualization of their treatment.

Furthermore, there is uncertainty about the optimal radiotherapy dose in esophageal carcinoma. For example, a phase III clinical trial documented that the survival or local/regional control in the group with a higher radiation dose (64.8 Gy) was not increased compared to that in the group with the lower radiotherapy dose (50.4 Gy) [5]. Moreover, in the CROSS phase III trial, disease-free survival (DFS) and overall survival were improved in the group that underwent preoperative chemoradiotherapy (CRT) compared with the group that underwent surgery alone [6].

However, traditional treatment evaluation systems are based on dimensional changes in tumour diameter or volume on anatomical MRI. Recent advancements in functional magnetic resonance imaging (MRI) techniques have the potential to be applied in the early assessment of treatment response in many cancers [7]. For example, diffusion weighted imaging (DWI) is a noninvasive approach to probe the molecular diffusion of water on the microscopic scale without contrast administration, and the apparent diffusion coefficient (ADC) value has been utilized to evaluate the treatment response to CRT in many cancers including esophageal carcinoma [8]. Nevertheless, only a few studies have evaluated the clinical significance of the capacity of DWI to predict the treatment response in patients with EC [9–11], and there is uncertainty about how to select the timing of observation post-therapy to avoid false negatives, and how to minimize the confounding factors that may interfere with the measurements [12].

It is well known that ADC values reflect the free diffusion of water molecules in a single voxel due to restrictive barriers such as membranes, macromolecules, and fibresin tissue compartments. Is the ADC value associated with some macromolecules? Aoyagi T [13] reported a negative correlation between the ADC of the tumours and the amount of vascular endothelial growth factor (VEGF). SIRT1, a member of the mammalian Sirtuin family, has been implicated to play vital roles in many biological processes including stress response, apoptosis, cellular metabolism, ageing, and a double effect on tumorigenesis [14]. In our previous study [15], we found that the expression level of protein Sirtuin-1 (SIRT1) was higher in CRT non-responder patients with ESCC than in CRT responder patients, and suppression of SIRT1 may inhibit the growth of ESCC cell lines. We also demonstrated that the downregulation of SIRT1 expression may inhibit the growth of ESCC cell lines, and that SIRT1 may be a biomarker for the treatment of EC [16]. With regard to ADC and SIRT1 as biomarkers in the treatment of EC, what is the relationship of both factors? To our knowledge, no published studies have determined whether the amount of SIRT1 in EC patients shows a biological correlation with the ADC value.

The goals of this study were to determine the correlation between the ADC values of patients with ESCC and the amount of SIRT1 in esophageal tumour tissue, to ascertain a better biomarker to predict the prognosis and therapeutic response for patients with ESCC.

## Methods

### Ethics statement

This study was approved by the Ethical Committee of Zhejiang Cancer Hospital. All patients were provided with and subsequently signed informed consent documentation.

### Patient database

Between January 2015 and December 2017, 75 patients diagnosed with ESCC at our hospital were enrolled in the study. All patients were qualified by the following criteria: (1). All cases were confirmed by pathology; (2). Without a second primary tumour and distant metastasis; (3). With normal liver/kidney function and routine blood examination and electrocardiograms; (4). Without any serious medical diseases; (5). Without any known history of chest CRT or surgery; (6). Eastern Cooperative Oncology Group (ECOG) performance status (PS) score ≤ 2; and (7). Without any contraindications for CRT. The exclusion criteria were as follows: (1). Contraindications for MRI examination; (2). With any signs of esophageal perforation in radiology examination including niche, penetrating ulcer, tortuosity, and angulation; or (3). With a medical history of systemic heart, liver, and kidney diseases. The classification of disease stage was according to the 7th edition of the Union for International Cancer Control (UICC) and the American Joint Committee on Cancer (AJCC) staging system.

### MR imaging

All subjects underwent two MRI examinations using a 3.0-T MR scanner (Achieva, Philips Medical Systems, Best, The Netherlands) equipped with a phased array body coil (SENSE body coil, Philips Medical Systems, Best, The Netherlands). Before the MRI examinations, all cases were arranged shallow and slow uniform breathing training to collect the required signals. The examinations were performed before the treatment and at the 5th fraction radiotherapy (RT). All MRI examinations contained T1 weighted imaging (TR/TE 423/100 ms, average number 1, FOV 365 × 284 mm, matrix 320, slice thickness 4 mm, skip 1.2–1.6 mm and slice 20), T2 weighted imaging (TR/TE 2000/70 ms, concatenations 2, flip angle 180°, matrix 288, average number 2, FOV 300 × 280 mm, slice thickness 4 mm, slice 20 and skip 1.2–1.6 mm), T1 plus contrast enhanced imaging including sagittal and transverse axial, and then DWI imaging (TR/TE/TI 10,205/70/180 ms, FOV 450 × 366 mm, matrix 256, slice thickness/gap 4/0 mm, slice 20, EPI factor 43). DWI scans were obtained using a single-shot spin-echo type of echo-planar sequence, and fat signals were suppressed using short-tau inversion recovery (STIR). The b-values of DWI were b = 0 and 1000 s/mm^2^.

### Imaging analysis

**Fig. 1:**
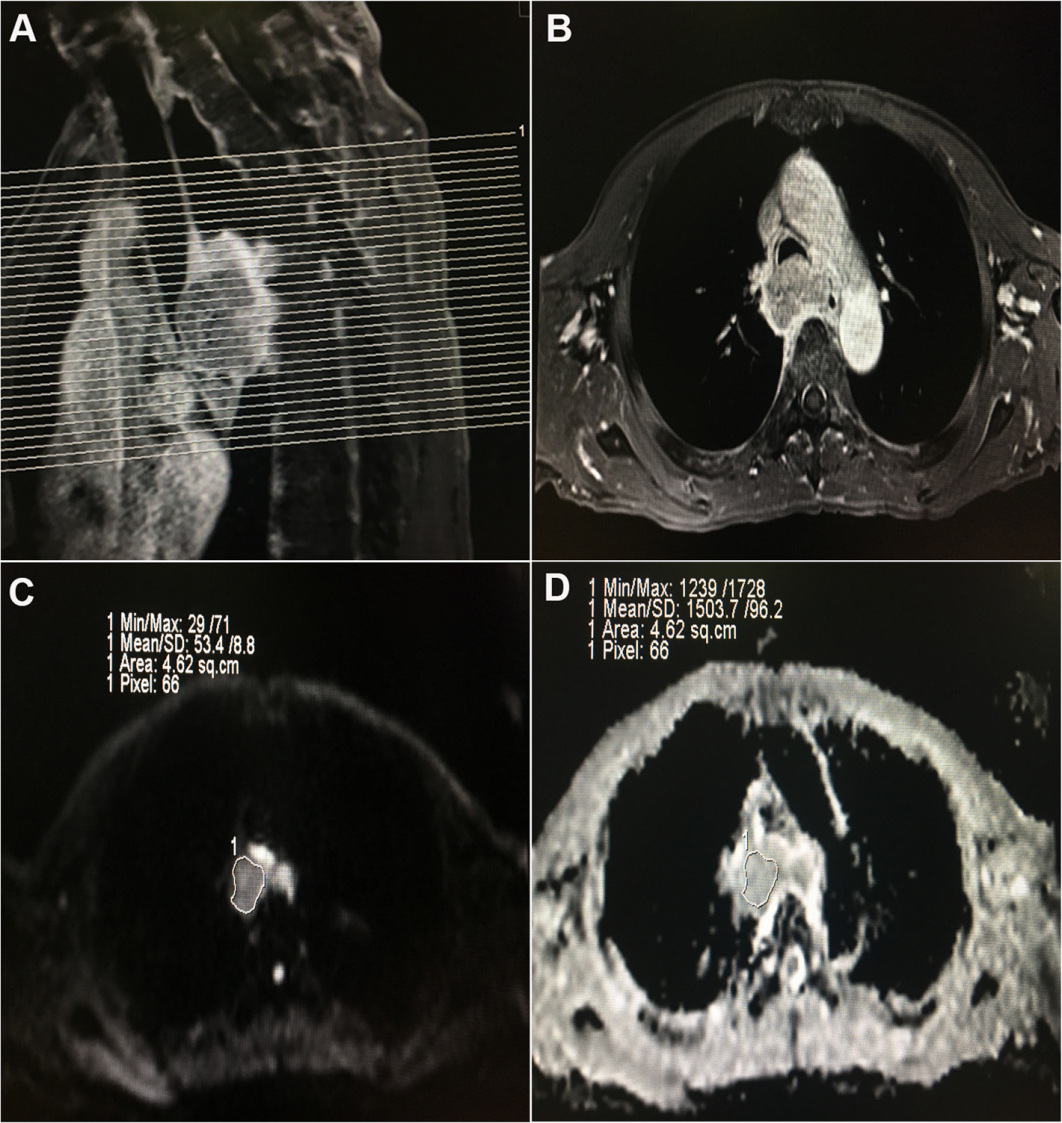
Example of ADC measurements of a primary tumour in a patient diagnosed with esophageal carcinoma before treatment. **a**. A sagittal view of T1 with contrast-enhanced (T1 + C) image with position line for the primary tumour. **b** A transverse view of the T1 + C image from the one of three continuous sections with maximal diameter based on the transverse and sagittal view. **c**. A region of interest (ROI) was contoured manually in the selected section of the DWI image, which was obtained at a b-value of 1000 s/mm^2^, and the ROI was then copied and pasted onto the ADC map (**d**), and the ADC value of the selected section was automatically calculated

Both MR images were transferred into a workstation (ViewForum; Philips Medical Systems, Best, The Netherlands). According to the images obtained from DWI, the corresponding ADC maps were obtained through DWI image fusion when the b value was 0 and 1000 s/mm^2^. The method of ROI contouring was depicted in the previous study, and three continuous slices centered in the largest and clearest regions on the lesion sites from the images of ADC maps were selected as the ROIs, and then three ADC values were automatically generated in ADC maps, which were averaged and labeled as ADC of tumour (Fig. 1). The pretreatment and intra-treatment ADCs were labeled as pre-ADC and intra-ADC respectively, and then ΔADC, and ADC_ratio_ were calculated according to the following equation: (intra-ADC - pre-ADC) and (intra-ADC - pre-ADC)/ pre-ADC, respectively.

### Analysis of SIRT1 levels in tumours

All patients undergoing the whole course of treatment were collected before treatment and then analysed for SIRT1 levels of tumours using immunohistochemistry (IHC). The primary antibodies to SIRT1 (rabbit pAb, Sigma antibody, USA, 1:200) were used, and then evaluated on the basis of the positively stained cell percentage and staining intensity. To quantitatively analyse the level of SIRT1, the tumour underwent imaging using light microscopy (Olympus, Tokyo, Japan) at a low magnification (× 40) to search for zones with strong positive staining. The staining zones were photographed at a magnification of × 400. IHC results were independently evaluated by 2 pathologists, and a score was recorded only when both pathologists agreed.

### Treatment regimens

All patients received definitive chemoradiotherapy (dCRT), and the chemotherapy regimen including docetaxel 75 mg/m^2^, intravenous (IV) bolus, day 1 and cisplatin 75 mg/m^2^, days 1 to 3 was administered every 3 weeks for 2 cycles. A prescription dose of 60.0 Gy was carried out in 30 fractions of 2.0 Gy per day, five fractions every week, starting at the first day of the first cycle of chemotherapy. The gross tumour volume was covered including the primary tumour and any enlarged regional lymph nodes, which were confirmed by all available information including computer tomography (CT), gastroscopy, and barium esophagram. All patients underwent intensity modulated radiotherapy (IMRT) technique.

### Follow-up

All patients were subject to a follow-up process, and the examinations including neck-thorax-abdomen contrast-enhanced CT, gastroscopy, and barium esophagram were appointed at 1 month after the treatment. The short-term response was based on the Response Evaluation Criteria In Solid Tumours 1.1 (RECIST version 1.1). Treatment response was subdivided into two groups: the Responder group and the Non-Responder group. The former included clinical complete response (cCR) and clinical partial response (cPR), and the latter included clinical stable disease (SD) and clinical progressive disease (PD).

### Statistical analysis

Pearson’s correlation coefficient was used to measure the association between ADC values and SIRT1 level. Spearman’s correlation coefficient was used to measure the association between ADC values and short-term response. The differences were considered significant when the *P* values were less than 0.05. All statistical analyses were performed using SPSS 16.0 software (SPSS, Chicago, IL, USA). The diagnostic accuracy of ADC regarding the predicting sensitivity in short-term response was analysed in concert with the receiver operating characteristic (ROC) curve. Continuous variables are expressed as the mean ± SD.

## Results

### The baseline characteristics of patients with EC between the responder and non-responder groups

Among the 75 patients with EC in the study, 66 patients fulfilled the entire course of treatment, and 9 patients were excluded because of the termination of treatment and drop-out. Forty-five patients were in the Responder group, while 21 patients were in the Non-Responder group. The number of patients in each T stage was T3, *n* = 52, T4, *n* = 14. Primary lesion sites were located in the cervical segment, upper thoracic, middle thoracic and lower thoracic. Twenty-four patients scored performance status (PS) of 0, 16 cases scored PS of 1, and 12 cases scored PS of 2. The mean prescription RT dose was 6060.75 ± 557.74 cGy. The details were recorded in Table 1.

**Table 1:** General clinical data of 66 esophageal carcinoma according to short-term response

Characteristics	Short-term response		*P* value
	Responder	Non-responder	
No.	45	21	
Age (years)	52.67 ± 9.82	49.45 ± 10.78	0.67
Gender			
Male	29	14	0.86
Female	16	7	
PS			0.71
0	19	11	
1	17	6	
2	9	4	
Location of tumor			0.18
Neck + Upper thoracic	11	8	
Middle thoracic	28	8	
Lower thoracic	6	5	
T stage			0.24
T3	38	14	
T4	7	7	
Mean prescriptive dose of RT (cGy)	6030.56 ± 480.96	6075.85 ± 645.58	0.58

### Variability of different ADC values according to short-term responses

**Fig. 2:**
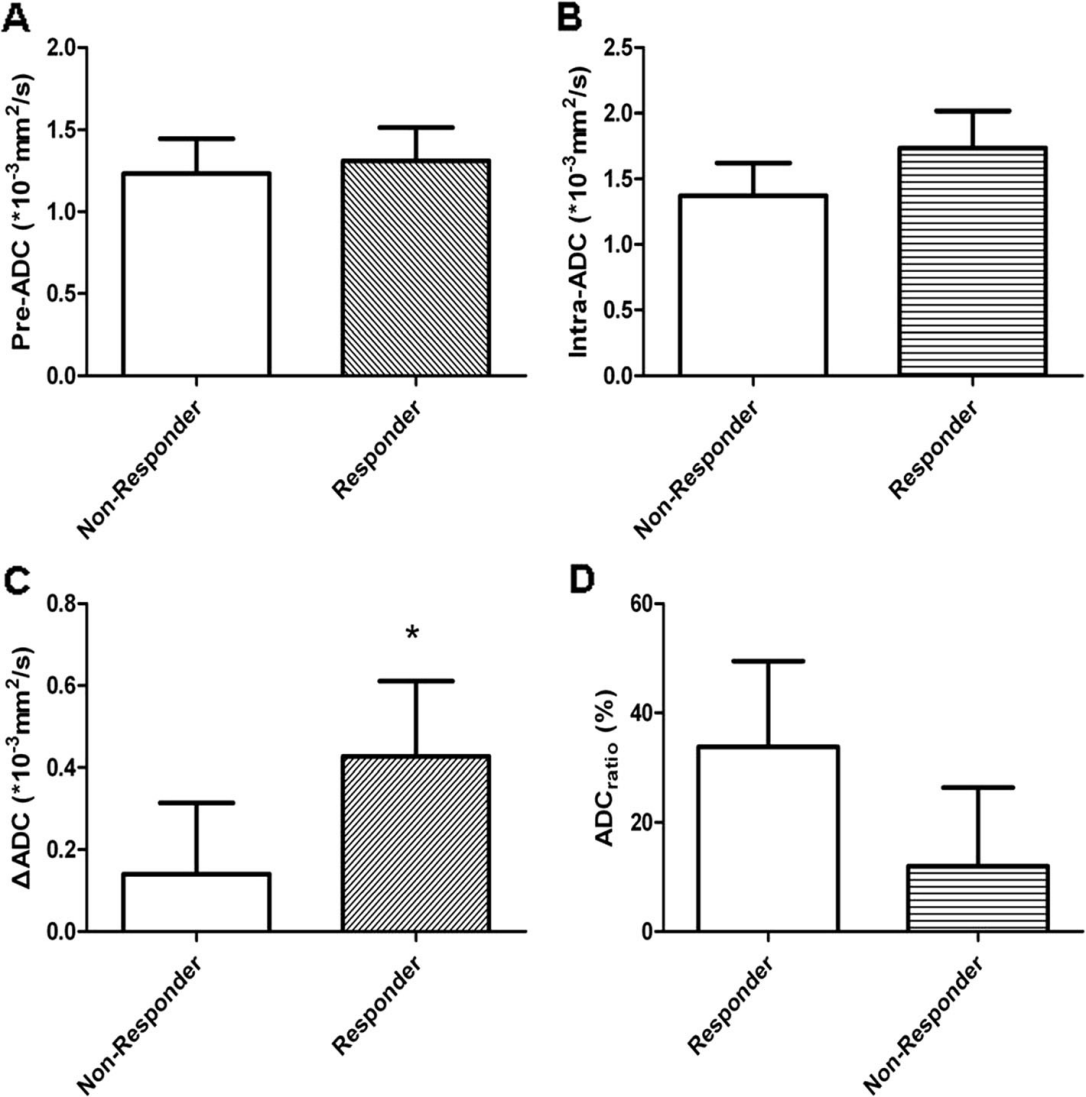
Comparisons of various ADC values including pre-ADC (**a**), intra-ADC (**b**), ΔADC (**c**) and ADC_ratio_ (**d**) according to different short-term responses. Only ΔADC showed significant statistical differences (*: *P* = 0.00, *P* < 0.05)

The mean pre-ADC and intra-ADC values of primary tumours in 66 cases were 1.29 ± 0.21 (10^− 3^ mm^2^/s) and 1.62 ± 0.32 (10^− 3^ mm^2^/s) respectively, and the mean ΔADC value was 0.34 ± 0.22(10^− 3^ mm^2^/s). There was a significant difference between pre-ADC and intra-ADC (*P* = 0.03). With regard to short-term response, mean pre-ADC, intra-ADC, ΔADC and ADC_ratio_ were 1.31 ± 0.20(10^− 3^ mm^2^/s) and 1.23 ± 0.21 (10^− 3^ mm^2^/s), 1.37 ± 0.25 (10^− 3^ mm^2^/s) and 1.74 ± 0.28(10^− 3^ mm^2^/s), 0.14 ± 0.17(10^− 3^ mm^2^/s) and 0.43 ± 0.18(10^− 3^ mm^2^/s), 11.98 ± 14.39(%) and 33.75 ± 15.72(%) in the Responder and Non-Responder groups respectively, and only ΔADC value showed significant differences (Fig. 2).

### Correlation and ROC analysis between ADC value and short-term response

**Fig. 3:**
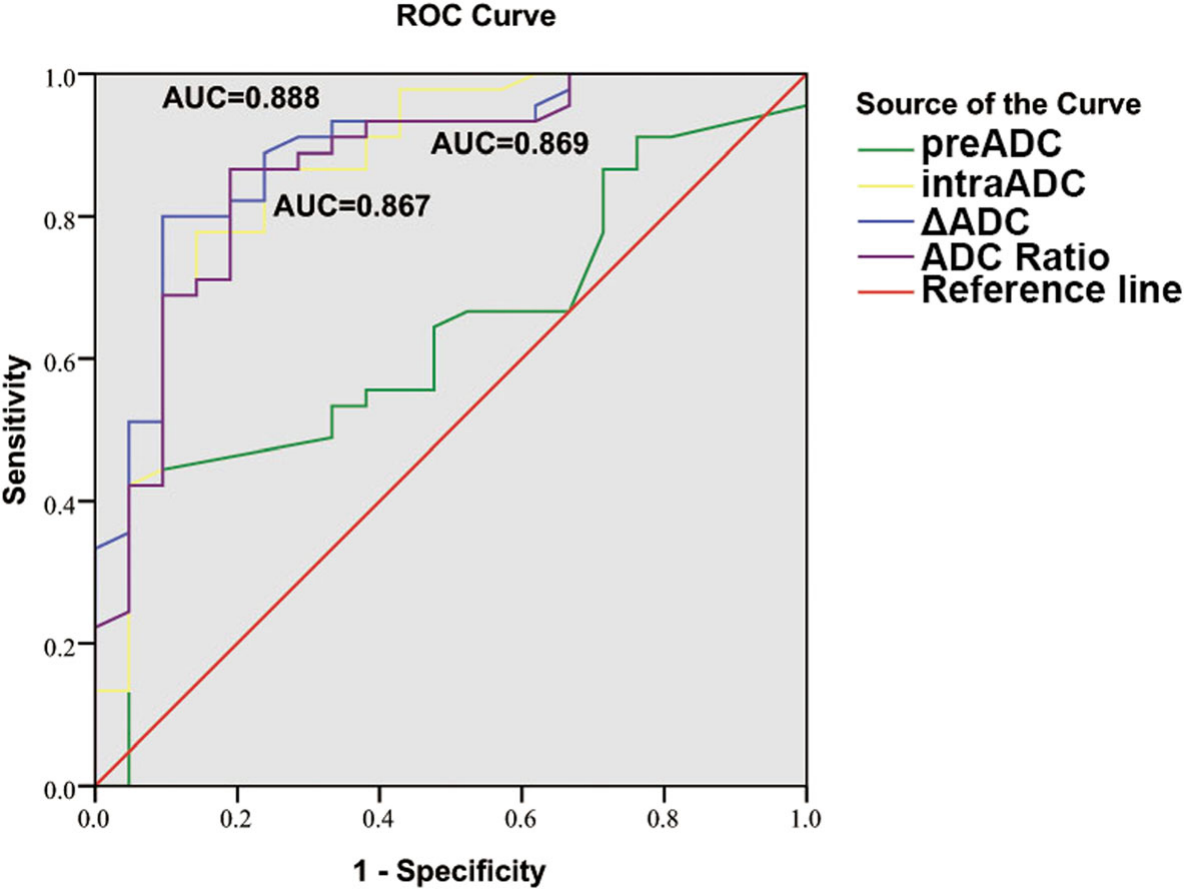
Constructing ROC curves of parametric ADCs to predict the accuracy of treatment response. AUC: area under the curve

Spearman’s rank correlation coefficient demonstrated that pre-ADC, intra-ADC, ΔADC and the ADC_ratio_ were positively correlated with the short-term response (*ρ* = 0.215, 0.595, 0.627 and 0.592 respectively, Table 2), and that only ΔADC may be an independent factor associated with the short-term response via logistic regression analysis (odds ratio: 875.03, 95%CI: 6.35~1.21E5). Constructing the ROC curve based on different responses, the area under the ROC curves (AUCs) for pre-ADC, intra-ADC, ΔADC and ADC_ratio_ were 0.633 (95%CI: 0.494~0.771, *P* = 0.084), 0.869 (95%CI: 0.767~0.970, *P* = 0.003), 0.888 (95%CI: 0.802~0.974, *P* = 0.001) and 0.867 (95%CI:0.769~0.965, *P* = 0.001), respectively (Fig. 3). According to the ROC curve, the diagnostic efficacy of short-term response was better evaluated at the point where ΔADC was at a threshold of 0.23*10^− 3^ mm^2^/s; the sensitivity was 82.2% and specificity was 81.0%.

**Table 2:** Spearman’s rank correlation coefficient of short-term response and ADC values

	Response	pre-ADC	Intra-ADC	ΔADC	ADC_ratio_	SIRT1
Response	1					
pre-ADC	0.215	1				
Intra-ADC	0.595^*^	0.331	1			
ΔADC	0.627^*^	−0.208	0.757^**^	1		
ADC_ratio_	0.592^**^	−0.359^*^	0.668^**^	0.968^**^	1	
SIRT1	−0.710^**^	−0.227	−0.749^**^	− 0.837^**^	−0.782^**^	1

**: *P* < 0.01 (2-tailed), *: *P* < 0.05 (2-tailed)

### Protein SIRT1 levels of primary tumours in EC patients using IHC examination

**Fig. 4:**
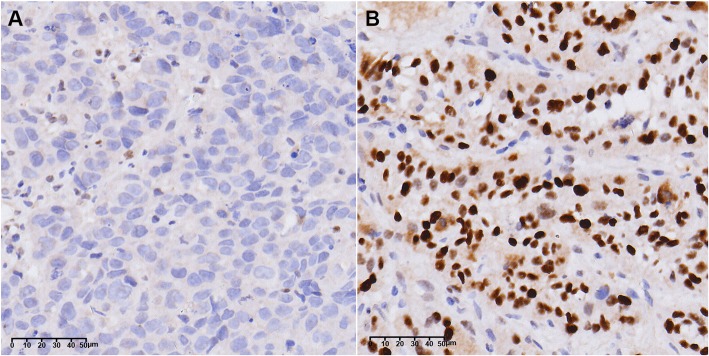
Expression of SIRT1 in EC patients before chemoradiotherapy using IHC. **a**. Negative/Low expression received CR in short-term response, **b**. Positive/High expression with brown staining, received SD in short-term response, × 400

The percentages of SIRT1 varied from 4.6 to 76.7%, and the mean percentage of protein SIRT1 in 66 EC patients was 36.6 ± 23.1 (%). Comparison of the SIRT1 levels between different short-term responses showed that the levels were higher in the Non-responder group (61.1 ± 11.9(%)) than in the Responder group (25.1 ± 17.3(%)) (Fig. 4), and Spearman’s rank correlation coefficient showed a negative correlation between the SIRT1 levels of the tumours and the short-term response (*ρ* = − 0.710, *P* < 0.01).

### Correlation analysis between ADC values and SIRT1 levels

**Fig. 5:**
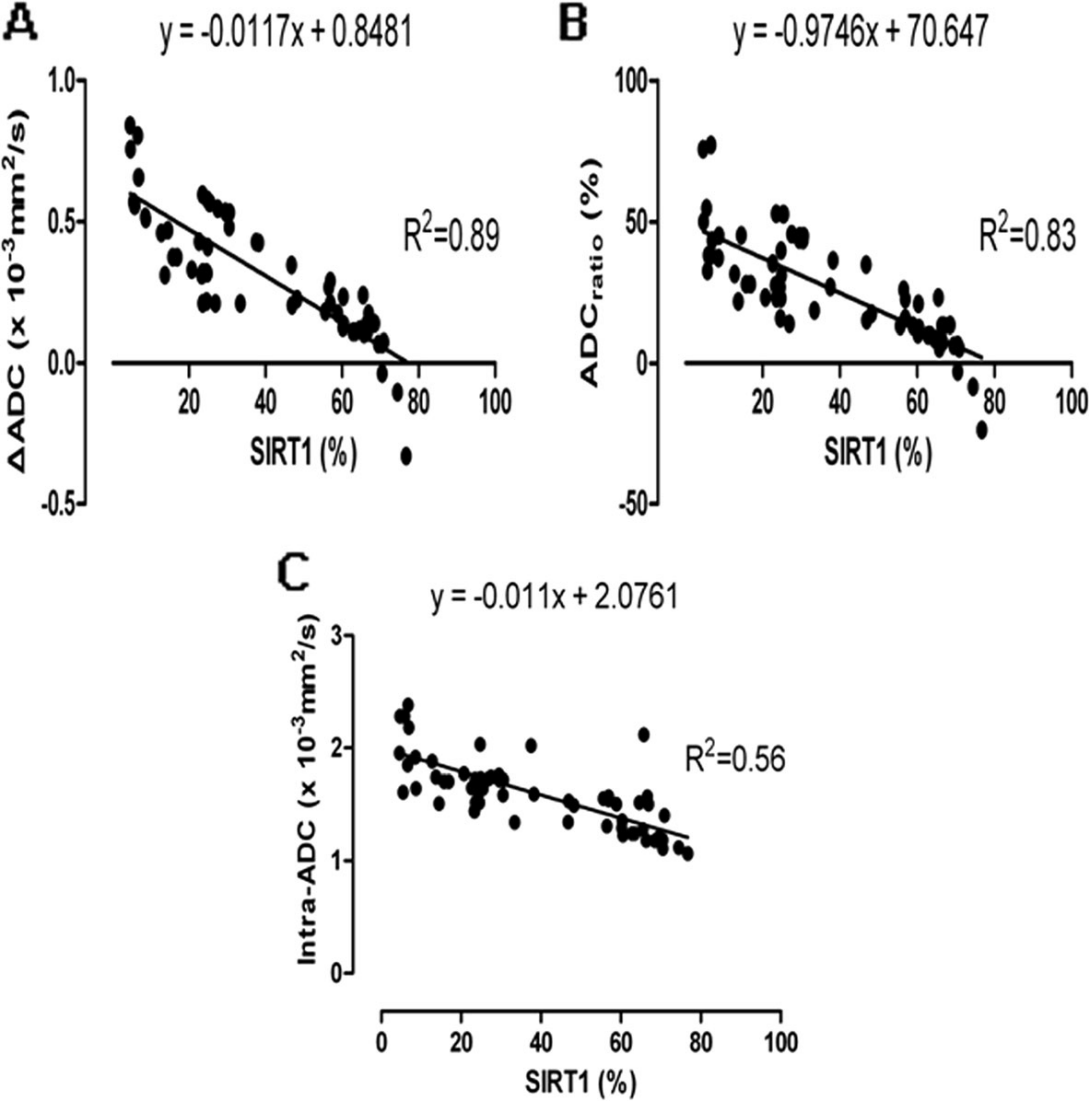
The correlation between the apparent diffusion coefficient (ADC) values and SIRT1 levels. The ΔADC values (**a**), ADC_ratio_ (**b**) and intra-ADC (**c**) (vertical axis) are plotted against the expression of the percentage of SIRT1 (horizontal axis). The circles indicate each ADC value as the percentage of SIRT1

Intra-ADC, ΔADC and ADC_ratio_ had various strong negative correlations with SIRT1 levels (intra-ADC: *r* = − 0.748, *P* = 0.002; ΔADC: *r* = − 0.943, *P* = 0.002; ADC_ratio_: *r* = − 0.911, *P* = 0.000), while a weak positive correlation between the pre-ADC and the levels of SIRT1 was observed, and no significant difference in the statistics was found (*r* = 0.109, *P* = 0.558) (Fig. 5).

## Discussion

In conventional evaluation systems, a decrease in tumour size may serve as a biomarker of treatment response, unfortunately, factors such as edema, necrosis, and inflammation can lead to anatomical distortion and signal changes difficult to distinguish from tumour residue during the course of CRT. In the current study, we endeavored to elaborate the biological correlation between ADC values and SIRT1 protein levels in tumour tissues in patients with ESCC, because SIRT1 was documented to be related to chemoradiation resistance by several studies including our preliminary studies [16–18]. Therefore, we illustrate ADC as a biomarker to predict the early response of CRT in EC patients.

We found that changes in both the ADC values (ΔADC) and the percentages of ADC (ADC_ratio_) in the Responder group were higher than those in the Non-Responder group in the early period of definitive CRT, and the rapid changes at the early point are more straightforward and may potentially be used to predict CRT responses well in advance. We propose that the acute increase of ADC indicates and acute decrease of cell density in EC patients sensitive to CRT such as the Responder group, mostly likely due to the death of tumour cells with an enlarged intercellular space caused by definitive CRT, and the different sensitivity in EC patients may be observed by early DWI examination. Furthermore, Spearman’s correlation analysis revealed that different ADC values except pre-ADC may have different strong positive relationships with the short-term response of CRT, and then multivariable logistic regression analysis revealed that only ΔADC may be an independent factor associated with the short-term response. Moreover, according to the results of ROC curve analysis, ΔADC was superior in identifying treatment response at the early time-point of CRT with a cutoff value of 0.23*10^− 3^ mm^2^/s and an AUC of 0.888 compared with other ADC values.

There are scarce reports on the ADC value as a biomarker in predicting tumour response prediction; however, the timing of the observation during CRT was uncertain and the cut-off value to predict the response has scarcely been reported in previous studies. In a previous animal model of rat brains implanted with 9 L glioma cells through gene therapy, ADC maps acquired over 10 days showed an increase in ADC values within the tumours by 31% after 8 days of treatment that preceded tumour growth arrest and regression [19]. In another pre-clinical study DWI in rabbits with metastatic tumour underwent irradiation, and 3 and 7 days after therapy, the ADCs were significantly higher than pretreatment and 1 day after therapy [20]. The above studies revealed that DWI is superior to morphological MRI in monitoring early radiation response in animal models. A multicenter trial using diffusion-weighted MRI findings to predict pathologic response in neoadjuvant treatment of breast cancer, change in breast tumour ADC (ΔADC) after 12 weeks of chemotherapy at MRI predicts complete pathologic response to neoadjuvant chemotherapy [7]. However, the correlated studies carried out in the field of esophageal carcinoma are sparse. Recently, a pilot study in EC patients showed that ADC changes at the time-point of 2–3 weeks after the start of CRT in the CR group were significantly higher than those in the PR group using intravoxel incoherent motion (IVIM) MRI, which indicated that IVIM parameters were useful in assessing the response to definitive concurrent CRT for EC patients, but the ADC was not performed at the early course of CRT [21]. Wang et al. [11] reported that the ADC values of ESCC increased gradually with the progression of concurrent CRT, and only the ADC values at Week 3 (15 fractions) were an independent prognostic factor of tumour response. The above studies, including our research, support the viewpoint of ADC change as a biomarker to predict the response of CRT in EC patients with differences at the time-point of observation. However, the study by Giganti et al. [22] showed that pretreatment ADC values below or equal to 1.4*10 − 3 mm^2^/s can predict a negative prognosis for esophageal cancer, and a study [23] from Japan showed that ADC values of pretreatment may be a useful marker to predict CRT response and high ADC values indicated a good response. Therefore, it is questionable at which time point and to what extent the evaluation of the target lesions may be valuable for further EC patient management. Multicenter prospective studies may solve this question.

Changes in the ADC value may indicate changes in the tumour inner structure by detecting diffusion changes in water, but the correlation between ADC and molecular pathologic information in tumour tissues remains unclear. To our knowledge, few studies have reported the correlation between ADC value and radioresistant-related protein. A study by Aoyagi T [13] et al. previously showed a negative correlation between the ADC of the tumours and the levels of VEGF, while protein VEGF was reported to be responsible for the malignant potential and a useful marker in estimating patient prognosis in esophageal carcinoma [24, 25]. Therefore, the ADC value may be a novel prognostic factor and contribute to the treatment of esophageal cancer by predicting the overexpression of VEGF protein. Previous studies revealed that aberrant expression of SIRT1 has been noted in many solid tumours including esophageal carcinoma, and the overexpression of SIRT1 is responsible for radiation resistance and poor prognosis in patients with esophageal cancer treated with chemotherapy and radiotherapy [18, 26]. In our previous study, we also revealed that SIRT1 was highly expressed in EC patients and that SIRT1 downregulation may inhibit the cell growth of ECSS cell lines [16]. In the current study, we compared the SIRT1 level between the Non-responder group and the Responder group. The level of SIRT1 in the latter group was significantly lower than in the former group, which is inverse with the correlation between ADC and short-term response. We further found a negative correlation between the change of ADC values (ΔADC) and the level of SIRT1. A possible explanation for this finding is that the changes of the ADC (ΔADC) are associated with the tissue cellularity/cellular density, while the levels of SIRT1 directly associated with chemoradiation resistance of tumour cells, also reflect the situation of cellularity/cellular density. A better understanding of the biological correlation between ADC and protein SIRT1 holds the promise of discovering predictive and prognostic biomarkers that might be helpful in the management of esophageal carcinoma.

Our study had several limitations. First, the sample size was relatively small, without variability in age, gender, and other factors. Second, further high-level evidence such as prospective multicenter trials are needed to verify the value of ADC in predicting the response of CRT in EC patients. Third, we did not analyze the situation of ADC in metastatic nodes. To overcome these limitations, a large number of patients will be enrolled in the prospective study to assess the predictive value of ADC in the primary site and metastatic nodes.

## Conclusion

In summary, the early increases in ADC may be used as a biomarker to predict the early response of CRT in patients with ESCC, and the high accuracy of ADC may facilitate the timely selection of patients for regimen adjustment. The reason may be partly explained by the different correlation between the change of ADC and the expression in SIRT1. Further prospective studies with a larger patient population are required to clinically validate these results.

## Data Availability

Raw data may be available on request from the corresponding author.

## Abbreviations

ADC: Apparent diffusion coefficient
AJCC: The American Joint Committee on Cancer
CR: Complete response
CRT: Chemoradiotherapy
DFS: Disease-free survival
DWI: Diffused weighed imaging
EC: Esophageal carcinoma
ECOG: Eastern Cooperative Oncology Group
ESCC: Esophageal squamous cell carcinoma
FOV: Field of view
IHC: Immunohistochemical
IMRT: Intensity modulated radiotherapy
IVIM: Intravoxel incoherent motion
MRI: Magnetic resonance imaging
PD: Progressive disease
PR: Partial response
PS: Performance status
RECIST: Response Evaluation Criteria In Solid Tumours
ROI: Regions of interest
RT: Radiotherapy
SD: Stable disease
SIRT1: Sirtuin1
STIR: Short-tau inversion recovery
UICC: Union for International Cancer Control
